# Enhancement of the Antiobesity and Antioxidant Effect of Purple Sweet Potato Extracts and Enhancement of the Effects by Fermentation

**DOI:** 10.3390/antiox10060888

**Published:** 2021-05-31

**Authors:** Seul Gi Lee, Jongbeom Chae, Dong Se Kim, Jung-Bok Lee, Gi-Seok Kwon, Taeg Kyu Kwon, Ju-Ock Nam

**Affiliations:** 1 Department of Immunology, School of Medicine, Keimyung University, Daegu 42601, Korea; lsg100479@naver.com (S.G.L.); kwontk@dsmc.or.kr (T.K.K.); 2 Center for Forensic Pharmaceutical Science, Keimyung University, Daegu 42601, Korea; 3 Department of Food Science and Biotechnology, Kyungpook National University, Daegu 41566, Korea; chejongbum@naver.com (J.C.); aodydirk@naver.com (D.S.K.); 4 Kyochon Research & Innovation Center, Kyochon F&B Co., Ltd., Chilgok-gun 18469, Korea; bio91@andong.ac.kr; 5 Department of Medical Plant Resources, Andong National University, Andong 36729, Korea; gskwon@andong.ac.kr; 6 Institute of Agricultural Science & Technology, Kyungpook National University, Daegu 41566, Korea

**Keywords:** 3T3-L1 adipocyte, antiobesity, browning, fermentation, purple sweet potato, transdifferentiation

## Abstract

The browning of white adipocytes, which transforms energy-storing white adipocytes to heat-producing beige adipocytes, is considered a strategy against metabolic diseases. Several dietary compounds, such as anthocyanins, flavonoids, and phenolic acids, induce a brown adipocyte-like phenotype in white adipocytes. In this study, we demonstrated that purple sweet potato (Ipomoea batatas) extract (PSP) exhibited potent radical scavenging activity. In addition, PSP was found to contain large amounts of phenolic, flavonoid, and anthocyanin compounds; the amount of these compounds was affected by fermentation. Functionally, PSP-induced adipose browning in high-fat-diet (HFD)-induced obese mice. The administration of PSP significantly suppressed the body weight gain and abnormal expansion of white adipose tissues in the obese mice. The expression of adipose browning-related genes was higher in the inguinal white adipose tissues from the PSP-treated mice than those in the HFD-fed mice. Moreover, PSP-treated 3T3-L1 adipocytes formed multilocular lipid droplets, similar to those formed in the 3T3-L1 adipocytes treated with a browning induction cocktail. The PSP-treated cells had an increased expression level of mitochondria and lipolysis-related genes. The browning effects of PSP were enhanced by fermentation with Lactobacillus. This study, to our knowledge, is the first to identify a new mechanism to increase the antiobesity effects of PSP by inducing adipocyte browning of adipocytes.

## 1. Introduction

Patients affected by obesity have excessive white adipose tissues (WATs), which play a role in energy storage as triglycerides (TGs) [1]. In contrast, brown adipose tissues (BATs) dissipate energy as heat in a process called thermogenesis through mitochondrial uncoupling. Thermogenesis is an appealing target for treating obesity, diabetes, and other metabolic disorders [2]. Since the metabolic activity of BATs is lower in patients affected by obesity, browning, or the induction of the brown adipocyte-like phenotype in white adipocytes, is considered a promising strategy for the treatment of obesity [3,4]. 

Several dietary compounds, such as curcumin, capsaicin, berberine, and some anthocyanins, have been proposed to be used to induce browning in mammals [5,6,7]. Purple sweet potato (PSP)—a functional food rich in anthocyanins—has many potential biological and pharmacological functions [8]. The anthocyanins from PSP are more stable than those in other plants such as strawberries, red cabbage, and perilla [8,9]. In addition, PSP exhibits anti-inflammatory and antiobesity effects [9,10]. Specifically, PSP exerts lipolytic effects on 3T3-L1 adipocytes by increasing the level of HSL and perilipin [10]. Lipolysis also occurs in brown adipocytes for mitochondrial β-oxidation and the activation of UCP1 that triggers thermogenesis [11]. However, the browning effects of PSP have not yet been investigated.

On the other hand, fermentation can modify the levels of most bioactive compounds, thus, promoting the utilization of value-added functional foods [12,13]. Microbial fermentation is a potential means of producing natural compounds causing various pharmacological activities, including antioxidant and antiobesity effects. The relationship between these activities has been supported by numerous studies [14,15]. For example, the antioxidant activity of fermented garlic by L. plantarum BL2 is enhanced compared to nonfermented garlic, exerting robust antiobesity effects on high-fat-diet (HFD)-induced mice [16]. Considering the association between the pharmacological activities and fermentation of compounds, we hypothesized that fermentation could improve the antioxidant and antiobesity effects of PSP. Here, we demonstrated the antiobesity effects of PSP on HFD-induced obese mice. We also evaluated the effect of PSPs on inducing the brown adipocyte-like phenotype in white adipocytes. We found that this effect was higher with fermented PSPs than nonfermented PSPs.

## 2. Materials and Methods

### 2.1. Sample Preparation

PSPs (Andong City Agricultural Technology Center, Andong, Korea) were extracted with 70% ethanol, lyophilized, reconstituted with PBS, and stored at 4 °C for use in the animal study. Different fermentation methods were used to prepare three different types of fermented PSP. During Lactobacillus fermentation (LF), PSP was cultured using lactic acid bacteria isolated from traditional Korean fermented foods for 3 days. During non-natural fermentation, PSP was inoculated with 7.0 ± 0.2 × 1012 CFU/mL Lactobacillus and cultured in broth at a ratio of 1:1 (*w/v*). During natural fermentation, PSP was fermented at 37 °C for 7 days. For each sample, 200 g was extracted three times in two volumes of 70% ethanol, concentrated under a vacuum to recover the samples, and stored at −20 °C. Later, the samples were dissolved in DMSO at specific concentrations and used for experiments. The nonfermented, nature fermented, and Lactobacillus-fermented PSP extracts were referred to as nonfermentation (NNF), natural fermentation (NF), and LF, respectively.

### 2.2. Animals and PSP Administration

The animal procedures were approved by the animal ethics committee at Kyungpook National University, Daegu, South Korea (Approval number: KNU 2020-0078). Six-week-old male C57BL/6 mice (Hyochang Science, Daegu, Korea) were kept in cages under a 12-h light/dark cycle at 25–30 °C for 1 week for acclimatization. The effect of PSP on HFD-induced obesity was examined by randomly dividing the mice into three diet groups, normal diet (ND), HFD, and HFD supplemented with PSP at 100 mg/kg/day (the PSP group). PSP was administered orally to the mice daily during the study, whereas an equal volume of sterile water was administered to the control mice. All the mice had ad libitum access to sterile water and corresponding diet food. The body weight and food intake of the mice were recorded weekly. At the end of the experiment, organs and blood were collected from each mouse for subsequent analysis.

### 2.3. Histological Analysis

Histological analysis was performed as previously described [17,18]. The livers and inguinal WAT (iWAT) of the mice were embedded in paraffin, sliced into 5-μm sections, stained with hematoxylin and eosin (H&E) according to standard protocol, and examined under a microscope (Leica, Wetzlar, Germany).

### 2.4. Measurement of Plasma Cholesterol

Plasma was prepared from whole blood via centrifugation. Total cholesterol (CHO) in the plasma was measured using an Olympus AU400 analyzer (Olympus Optical, Tokyo, Japan), according to the manufacturer’s instructions [19].

### 2.5. Glucose Tolerance Test

The glucose tolerance test was performed on 14-week-old mice and administered with PSP or sterile water. The mice were intraperitoneally injected with 1 g/kg D-glucose. The glucose levels were measured from the tail bleeds at 0, 15, 30, 60, 90, and 120 min after injection using an AccuChek-EZ glucose monitor (Roche Molecular Biochemicals, IN, USA).

### 2.6. Cell Culture and Differentiation

Preadipocyte 3T3-L1 cells (Korea Cell Line Bank, Seoul, Korea) were maintained in Dulbecco’s modified Eagle medium (DMEM; GIBCO, Grand Island, NY, USA) supplemented with 10% bovine calf serum (BCS; GIBCO). Differentiation was induced by culturing the 3T3-L1 preadipocytes to postconfluence (designated as Day 0) for 2 days and replacing the media with a differentiation induction medium consisting of 0.5 mM 3-isobutyl-1-methylxanthine, 0.25 μM dexamethasone, 0.125 mM indomethacin, and 1.72 nM insulin for 2 days. Afterward, the cells were maintained in media supplemented with 1.72 nM insulin for 6–7 days. For the brown adipocyte-like induction of 3T3-L1, we added a browning cocktail (BC) of 50 nM triidothyronine and 1 μM rosiglitazone to the differentiation induction medium. The cytotoxicity of the PSPs was evaluated by treating the 3T3-L1 preadipocytes with or without PSP for 24 h before conducting the MTT assay, as described previously [19].

### 2.7. Oil Red O Staining

Oil Red O staining (ORO) was performed as previously described [20]. The cells were washed, fixed, and stained with an ORO solution (Sigma-Aldrich, St. Louis, MO, USA). The level of staining was quantified by dissolving the stained cells in isopropyl alcohol and measuring the absorbance at 495 nm.

### 2.8. Real-Time Reverse Transcription Polymerase Chain Reaction (RT-PCR)

The isolation of total RNA, synthesis of complementary DNA (cDNA), and reverse transcription polymerase chain reaction (RT-PCR) were performed as previously described [20]. Total RNA was isolated from 3T3-L1 adipocytes and iWAT using the RNAiso Plus reagent (TakaRa Bio, Shiga, Japan). Then, cDNA was synthesized using the PrimeScript™ RT Reagent Kit (TaKaRa Bio). Quantitative RT-PCR was conducted with the iCycler iQ™ Real-Time PCR Detection System (Bio-Rad Laboratories, Hercules, CA, USA) using SYBR Green (TOYOBO, Osaka, Japan) and specific mouse primers (Table 1). The fold change in the target gene was normalized to the level of β-actin. 

### 2.9. Western Blot Analysis

The tissues and cells were lysed with radioimmunoprecipitation assay (RIPA) lysis buffer (Biosesang, Korea), homogenized, and centrifuged at 13,000 rpm for 15 min. Western blotting was performed [18,21] using anti-PGC-1α and anti-β-actin antibodies (Santa Cruz Biotechnology, Santa Cruz, CA, USA).

### 2.10. Measurement of Total Polyphenolic, Flavonoid, and Anthocyanin Contents

#### 2.10.1. Total Phenolic Content

The total phenolic content (TPC) of the PSPs was determined using Folin–Ciocalten’s reagent [22], as described [23]. A volume of 100 μL of RAE was mixed with 50 μL of Folin–Ciocalten’s reagent and 300 μL of 2% Na2CO3. After 15 min, 1 mL of distilled water was added, and the absorbance was measured by spectrophotometry (UV-2550, Shimazu Co., Tokyo, Japan) at 725 nm. The results were expressed as mg gallic acid equivalent (mg GAE/g) based on a standard curve (R^2^ = 0.9987), using gallic acid standards of 25–250 μg/mL.

#### 2.10.2. Total Flavonoid Content

The total flavonoid content (TFC) was analyzed using the aluminum colorimetric assay as described by Teng et al. [24]. A volume of 70 μL of the extract was diluted with 430 μL of distilled water, mixed with 50 μL of 5% NaNO2 and 50 μL of 10% Al(NO3)3.9H2O. After incubation at room temperature for 6 min, 500 μL of 1 N NaOH was added, and the absorbance was measured by spectrophotometry at 510 nm. The results were expressed as mg of rutin equivalent per mL (mg RE/mL) based on a standard curve (R^2^ = 0.9984) using rutin standards of 0.8–2.0 mg/mL.

#### 2.10.3. Total Anthocyanin Content

The TFC was determined using the pH differential method. The PSPs were diluted with 500 μL of 0.025 M hydrochloric acid-potassium chloride (pH 1.0) or 500 μL of 0.4 M sodium acetate (pH 4.5), incubated for 15 min at room temperature. The absorbance of the low- and high-pH samples was measured at 530 and 700 nm using a UV-Vis spectrophotometer, respectively. The total anthocyanin content was expressed as cyanidin-3-glucoside equivalents in the following equation:

TAC (mg chanidin-3-glucodise/L) = (A × 445 × DF)/26900(1)

where A = (A530 − A700 nm)pH 1.0 − (A530 − A700 nm)pH4.5 and DF denotes the dilution factor of the sample.

### 2.11. Antioxidant Analysis

The antioxidant activity of PSPs was determined using the 2,2-diphenyl-1-picrylhydrazyl (DPPH) and 2,2’-azino-bis(3-ethylbenzothiazoline-6-sulfonic acid) ABST radical scavenging methods [25]. The DPPH reagent at 100 mM was prepared by reacting 0.2 mM DPPH in 100 mL 95% ethanol for 2 h. Then, 100 μL of the DPPH extract was mixed with a 900-μL DPPH solution dissolved in ethanol; 95% ethanol served as the control. The sample was kept in the dark for 30 min, and absorbance was measured by spectrophotometry at 517 nm. DPPH radical scavenging activity was calculated in percentages using the following formula:

DPPH (%) = (control absorbance − sample absorbance/control absorbance) × 100(2)

On the other hand, the ABTS radical solution was prepared by reacting 7 mM ABTS and 2.45 mM aqueous potassium persulfate with 10 mL of distilled water in the dark at room temperature for 16 h. Next, the absorbance at 734 nm was adjusted to approximately 1.0 using distilled water. Then, 50 μL of the sample at 0.125, 0.25, 0.5, or 1 mg/mL was incubated with 950 μL of ABTS solution in the dark for 30 min. Ethanol was used in the control reaction. The absorbance was measured at 734 nm on a spectrophotometer (UV-2550, Shimazdu Co., Tokyo, Japan), and the ABTS scavenging capacity was calculated as follows:

ABTS scavenging capacity (%) = (control absorbance − sample absorbance/control absorbance) × 100(3)

### 2.12. Statistical Analysis

All data were expressed as means ± standard deviation (SD). The control and PSP-treated groups were compared using one-way ANOVA. A *p* value less than 0.05 was considered statistically significant.

## 3. Results

### 3.1. PSP Reduced Body Weight Gain and Suppressed Adipose Tissue Expansion in HFD-Induced Obese Mice

**Figure 1:**
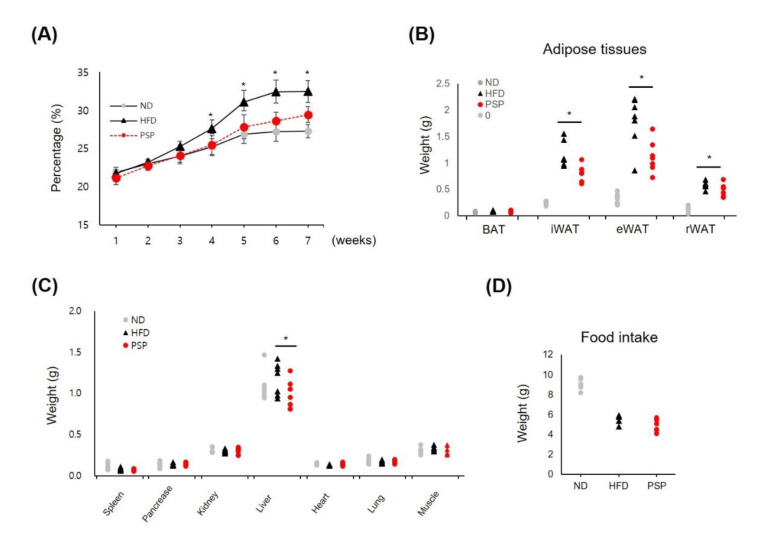
Effects of PSP on body weight gain, adipose expansion, and food intake in mice with HFD-induced obesity: The ND group (*n* = 7) was fed a normal diet, HFD control group (*n* = 7) was fed an HFD, and PSP-treated group (PSP) (*n* = 7) was fed an HFD plus PSP at 100 mg/kg/day. The ND and HFD control groups were given the same volume of water. (**A**) The body weight for 7 weeks. (**B**) The weight of adipose tissues, including BAT, iWAT, eWAT, and rWAT (*n* = 7/group). (**C**) The weight of the liver, heart, spleen, pancreas, lung, muscle, and kidney (*n* = 7/group). (**D**) Food intake was recorded every week throughout the study (*n* = 6/group). * *p* < 0.05, significant difference compared with HFD group. Bar graphs show mean ± SD of each group.

Initially, we studied the antiobesity effects of PSP on HFD-induced obese mice. The PSP-treated mice showed a significant decrease in body weight gain after 4 weeks of treatment (Figure 1A). The abnormal expansion of the WATs, including iWAT, epididymal WAT (eWAT), and retroperitoneal WAT (rWAT), and the liver were suppressed in the PSP-treated mice. In contrast, the BATs and other organs in the PSP-treated mice were similar to those in the HFD control mice (Figure 1B,C). Notably, food intake did not differ significantly between the PSP and HFD control mice (Figure 1D). These data indicate that PSP administration may affect the abnormal body weight gain and tissue expansion in the HFD-induced obese mice.

### 3.2. PSP Ameliorated the Metabolic Syndrome Associated with Obesity

**Figure 2:**
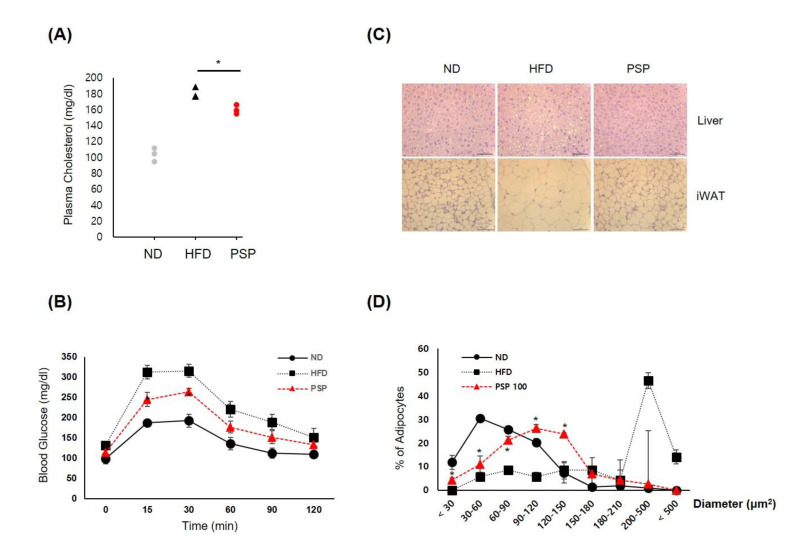
Effects of PSP on HFD-induced metabolic abnormalities: (**A**) The plasma CHO level of the mice in the indicated groups (*n* = 3/group). (**B**) Glucose tolerance test of the mice in the indicated groups. (**C**,**D**) The representative images of liver (upper) and iWAT tissues (lower) were stained with H&E (*n* = 3/group) (**C**). Adipocyte size was measured using the ImageJ software. (**D**) The density curves of the ND, HFD, and PSP groups. * *p* < 0.05, the significance of the difference from the HFD group. The bar graphs display the mean ± SD of the three mice per group.

We analyzed whether PSP administration affected the HFD-induced metabolic abnormalities, such as impaired lipid and glucose homeostasis. The PSP-treated mice had lower plasma CHO levels, with improved glucose tolerance (Figure 2A,B) and suppressed adipocyte hypertrophy and hepatic steatosis (Figure 2C,D) compared to the HFD control mice. Both adipocyte size and liver lipid deposition were smaller in the PSP-treated mice than in the HFD control mice (Figure 2C). In particular, the sizes of adipocytes in the adipose tissues of the PSP-treated mice were smaller than those in the HFD control mice (Figure 2D). On the other hand, the PSP and ND mice displayed similar patterns of adipocyte size distribution (Figure 2D).

### 3.3. PSP Induced Browning Features in Adipose Tissue

**Figure 3:**
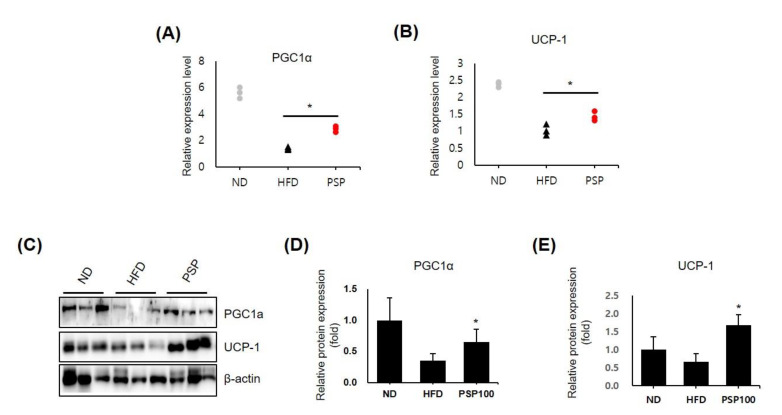
Effects of PSP in the expression of browning-related genes: (**A**,**B**) The mRNA levels of PGC1α (**A**) and UCP-1 (**B**) in the iWATs from the ND, HFD, and PSP mice (*n* = 3/group). The expression level of each gene was normalized to that of β-actin and expressed relative to the HFD control. (**C**–**E**) The Western blots (**C**) of PGC-1α and UCP-1 and the quantification of these proteins (**D**,**E**). * *p* < 0.05, the significance of the difference from the HFD group. The bar graphs show the mean ± SD of the three mice per group.

The results above suggested that adipose hypertrophy and hyperplasia were diminished in the PSP-treated mice; however, there was no difference in food intake between the PSP-treated and HFD control mice. Thus, we hypothesized that PSP administration accelerated energy expenditure through adipose browning. We observed the dramatically upregulated expression of browning-related genes, including PGC1α and UCP-1, in the iWAT in the PSP-treated mice compared to those in the HFD control mice (Figure 3A,B). Similarly, the protein levels of PGC1a and UCP-1 were increased in the adipocytes of the PSP-treated mice (Figure 3C–E). These observations indicate that PSP may regulate energy expenditure and protect against HFD-induced metabolic abnormalities by regulating these molecules.

### 3.4. The Influence of PSP in the Differentiation of 3T3-L1 Adipocytes

**Figure 4:**
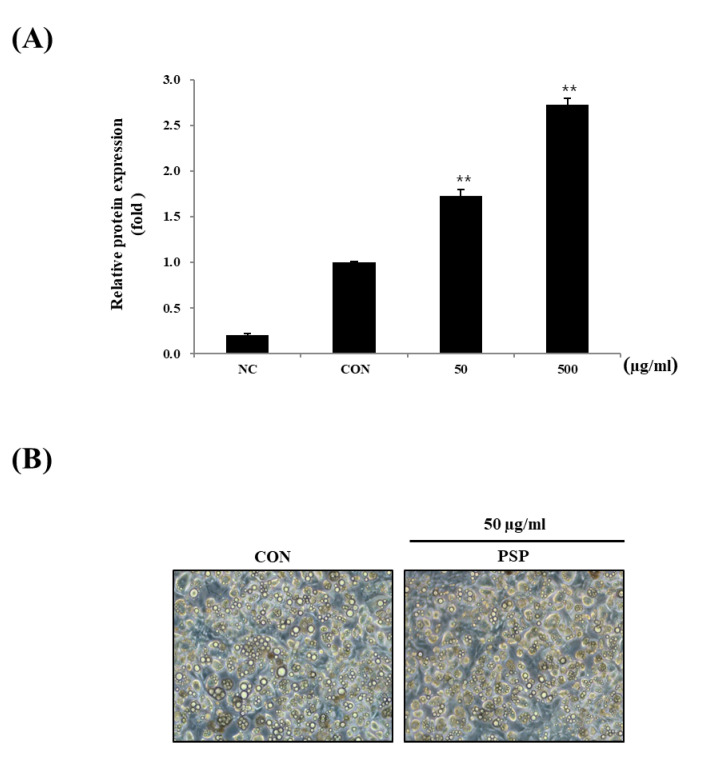
Effects of PSP on the differentiation of 3T3-L1 preadipocytes: (**A**) The expression of PPARγ in each experimental group. (**B**) A representative image of the control and PSP-treated cells at the end of the differentiation period (8–9 days). The cells were photographed under a microscope at 200 × magnification. Preadipocytes and mature adipocytes acted as the negative control (NC) and control (CON), respectively. All cell culture experiments are representative of three independent experiments. Data are presented as the mean ± SD of three independent experiments. ** *p* < 0.01, comparison to the control.

Next, we investigated PSP’s effects on in vitro adipocyte differentiation. The 3T3-L1 preadipocytes were treated with PSPs at 50 or 500 µg/mL during the entire period of differentiation. The PSP-treated cells displayed the increased expression of PPARγ—a key transcription factor of adipogenesis (Figure 4A). Contrary to our expectations, PSPs did not change the number of mature adipocytes. In general, we believe that the increased expression of PPARγ in mature adipocytes indicates excessive intracellular lipid accumulation and hyperplasia. Conversely, in recent years, there has been burgeoning evidence suggesting that PPARγ likewise coordinates with adipose browning through multiple signaling pathways [26,27]. One of the interesting findings in this study is that the PSP-treated cells showed small multilocular lipid droplets compared with control cells (Figure 4B). Based on these results, we hypothesized that PSPs could regulate adipocyte lipid metabolism and their properties as well as the expression of adipogenic marker genes.

### 3.5. PSPs Induce Brown Adipocyte-Like Phenotype in White Adipocytes

**Figure 5:**
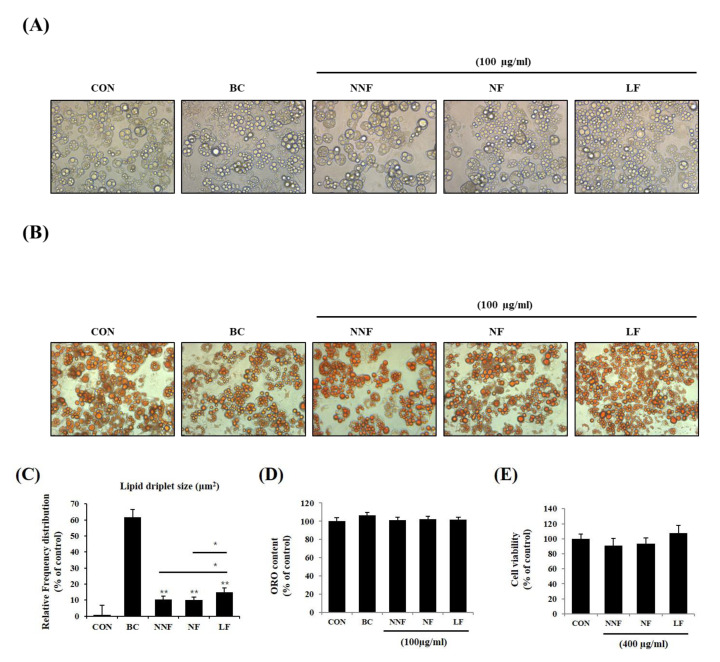
Effects of different fermented PSPs on the differentiation of 3T3-L1 preadipocyte: (**A**–**C**) The adipocytes were treated with NNF, NF, or LF at 100 µg/mL during the entire period of differentiation. BC, treating the 3T3-L1 adipocytes with a browning cocktail. The representative images of the cells (**A**) and ORO-stained-cells (**B**) in each indicated group. (**C**) Intracellular lipid droplets size was measured using ImageJ software. (**D**) The ORO staining in the cells was dissolved and measured. (**E**) The preadipocytes were treated with NNF, NF, or LF at 400 µg/mL or not treated (CON). After treatment for 24 h, cell viability was determined using the MTT assay. The bars represent the means ± SD of three independent experiments. *p < 0.05 and ** *p* < 0.01, comparison to the control.

We examined if different fermentations produced varying effects. We prepared three types of PSPs, nonfermentation (NNF), NF, and LF, at 100 µg/mL and administered them to the 3T3-L1 adipocytes. NNF-, NF-, and LF-treated cells displayed small intracellular sizes and multilocular lipid droplets compared to the control cells (Figure 5A,B). The morphology of NNF-, NF-, and LF-treated cells was similar to that of the BC-treated cells. Among the fermented PSP-treated cells, the LF-treated cells exhibited smaller lipid droplets, with the diameters in the range of 4-25 μm^2^, than the NNF- and NF-treated cells (Figure 5C). Consistent with the above results, the ORO-stained intracellular lipids in the PSP-treated cells were smaller than those in the control cells, although the total ORO-stained content did not vary between the control and PSP-treated cells (Figure 5D). We also did not observe any cytotoxic effects of NNF, NF, and LF on the 3T3-L1 preadipocyte at 400 µg/mL (Figure 5E). These results suggest that PSPs can change adipocyte cell size and lipid droplet morphology but not the total lipid content.

### 3.6. LF Increases mRNA Expression of Browning- and Adipogenesis-Related Genes in the 3T3-L1 Adipocytes

Mitochondrial content, which triggers lipolysis and decreases adipocyte TG content, is an important feature that defines the characteristics of brown, beige, and white adipocytes [28,29]. We attempted to uncover the possible function of PSPs on adipocytes by investigating the regulation of the expression of various mitochondrial genes, such as UCP-1, Tfam, Pdk4, and Sod2, and lipolysis-related genes, such as Atgl, Adrb3, and Srebf1, by the PSPs. 

**Figure 6:**
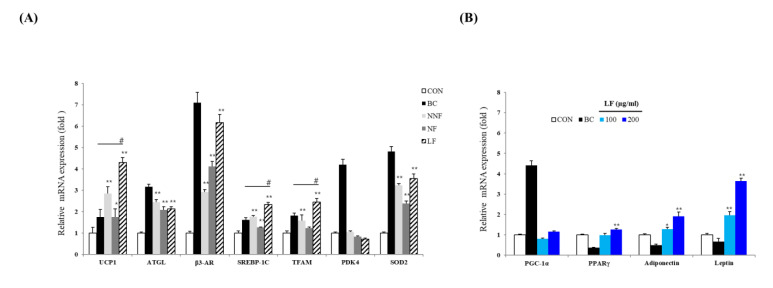
Effects of LF on the expression of adipogenic and thermogenic marker genes: (**A**) The mRNA level of UCP-1, ATGL, β3-AR, SREBP-1c, TFAM, PDK4, and SOD2 of each indicated group. The expression level of each gene was normalized to that of Actb and was expressed relative to the control. BC, treating the 3T3-L1 adipocytes with a browning cocktail. The bars represent the means ± SD of three independent experiments. * *p* < 0.05 and ** *p* < 0.01, comparison to the control. # *p* < 0.01, comparison to BC. (**B**) The expression of Ppargc1a, Pparg, Adipoq, and Lep. The mRNA level of each gene was normalized to that of Actb and expressed relative to the control. The bars represent the means ± SD of three independent experiments. * *p* < 0.05 and ** *p* < 0.01, comparison to the control.

The expression of UCP-1, ATGL, β3-AR, SREBP-1C, TFAM, and SOD2 in the PSP-treated cells was higher than that in the control cells (Figure 6A). Notably, the expression of UCP-1, Srebf1, and Tfam was increased most significantly in the LF-treated cells compared to the BC-treated cells. These results suggest that PSPs improve the lipolytic function and mitochondrial biogenesis in adipocytes, causing adipose browning. Moreover, these results indicate that fermented PSP may exert a more potent browning effect than unfermented PSP. Based on these results, we determine that the PSPs trigger the browning of 3T3-L1 adipocytes.

The expression level of several mitochondrial and lipolysis-related genes was significantly higher in the LF-treated cells than in the NNF- and NF-treated cells (Figure 6A). Thus, we further investigated LF regarding the browning effect. We examined whether treatment with LF regulated not only browning but also adipocyte differentiation. We confirmed the expression of a common adipogenesis marker and adipokine genes. The cells were treated with different concentrations of LF, and the expression of the genes was analyzed. The cells treated with LF at 200 µg/mL had an increased mRNA level of Pparg, Adipoq, and Lep compared to the control cells (Figure 6B). LF’s effects on the expression of adipokine genes, including Adipoq and Lep, were also observed in a dose-dependent manner. Moreover, the LF treatment increased the expression of Ppargc1a, although not in a statistically significant manner (Figure 6B).

### 3.7. Effects of PSPs on Antioxidant Activities

Finally, we examined the antioxidant activities of the PSPs using ABTS and DPPH radical scavenging assays. NNF, NF, and LF exhibited high scavenging activity with half-maximal inhibitory concentration (IC50) values of 0.92, 1.28, and 1.00 mg/mL, respectively, in the ABTS assay and 1.06, 1.56, 1.04 mg/mL, respectively, in the DPPH assay (Table 2). Since bioactive compounds, including phenolic, flavonoid, and anthocyanin compounds, generally possess higher antioxidant activity [30], we determined the contents of these compounds in the PSPs (Table 3). Notably, we found the highest levels of TPC and TFC in 200 ug/mL LF, at 7.89 ± 1.08 and 135.43 ± 1.45 GAE µg/mL, respectively. This result indicates that these bioactive compounds contribute to the antioxidant properties of PSPs.

## 4. Discussion

Herein, we have demonstrated for the first time that PSP improves HFD-induced metabolic abnormalities, including abnormal body weight gain, adipose hypertrophy, and impaired homeostasis. In addition, we have found that PSP increases the mRNA and protein levels of PGC1α and UCP-1 in WATs; therefore, the antiobesity effects of PSP may be mediated by adipose browning. The three types of PSP extracts tested here all triggered a change in adipocyte properties, transitioning white adipocytes to brown-like adipocytes and increasing the expression of the thermogenic marker genes. Among all the PSP extracts tested, we found that LF had a dramatic browning effect, as assessed by most mitochondrial- and lipolysis-related genes. These data suggest that bacterial fermentation is a good strategy for improving PSP’s antiobesity effects.

Our results are contrary to a previous observation that PSP exerts antiobesity effects by suppressing the expression of adipogenic genes and Lep in 3T3-L1 adipocytes [10]. We believe that such inconsistency may be due to the differences in experimental design, including the extraction process, PSP concentration used, and duration of the PSP treatment. In the previous study, the adipocytes were treated with PSP after the induction of adipocyte differentiation for 24 h [10]. In contrast, we treated the adipocytes with PSP throughout the differentiation period. Despite this inconsistency, the lipid droplet size in the PSP-treated cells was observed to be decreased in both studies. Most notably, we have identified a new method to increase the antiobesity effects of PSP via inducing adipocyte browning. 

Another significant finding is that the expression of common adipogenic genes, such as those encoding PPARγ, adiponectin, and leptin, was increased by LF treatment. PPARγ, a known master regulator of adipogenesis, is responsible for inducing the expression of adipogenic genes [31,32]. Although we did not provide direct evidence and the adipocytes were only analyzed at the end of differentiation, our results suggest that LF can influence initial-stage adipogenesis, not inhibit adipogenesis. On the other hand, microbial fermentation can often regulate the content of many active substances [33]. In this study, we have ascertained that fermentation can change the content of bioactive constituents, including TPC, TFC, and TAC, in PSP. This observation suggests that the differential antioxidant activity of PSPs likely results from their varying bioactive constituents.

## 5. Conclusions

In summary, our study has demonstrated that PSPs induce adipose browning and exert antioxidant action, thus protecting the mice from HFD-induced obesity. We also propose that LF is a good strategy for enhancing the antiobesity and antioxidant effects of PSPs. We thereby suggest that PSPs can be used as a dietary supplement to fight obesity and related oxidative stress.

## Figures and Tables

**Table 1:** Primer sequences for RT-PCR.

Gene Name	Accession No.	Forward Primer	Reverse Primer
**ATGL**	NM_001163689.1	AACGCCACTCACATCTACGG	GGACACCTCAATAATGTTGGCAC
**β3-AR**	NM_013462.3	CCTTCAACCCGGTCATCTACTG	CGCACCTTCATAGCCATCAAA
**SREBP-1c**	NM_011480.4	GCTGTTGGCATCCTGCTATC	TAGCTGGAAGTGACGGTGGT
**TFAM**	NM_009360.4	CAAAGGATGATTCGGCTCAG	AAGCTGAATATATGCCTGCTTTTC
**PDK4**	NM_013743.2	CCGCTGTCCATGAAGCA	GCAGAAAAGCAAAGGAC
**SOD2**	NM_013671.3	ACCTGCCTTACGACTATGGC	CCACCATTGAACTTCAGTGC
**PGC1α**	XM_006503778.3	CCCTGCCATTGTTAAGACC	TGCTGCTGTTCCTGTTTTC
**PPARγ**	AB644275.1	GGAAGACCACTCGCATTCCTT	GTAATCAGCAACCATTGGGTCA
**UCP-1**	NM_009463.3	CTGCCAGGACAGTACCCAAG	TCAGCTGTTCAAAGCACACA
**Adiponectin**	NM_009605.4	GATGGCACTCCTGGAGAGAA	TCTCCAGGCTCTCCTTTCCT
**Leptin**	NM_008493.3	GGGCTTCACCCCATTCTGA	TGGCTATCTGCAGCACATTTTG
**β-actin**	EF095208	CGTGCGTGACATCAAAGAGAA	GCTCGTTGCCAATAGTGATGA

**Table 2:** ABTS and DPPH antioxidant activity. Data are expressed as means ± SD (*n* = 3).

		IC50 (mg/mL)
**ABTS assay**	Ascorbic acid (standard)	0.0687 ± 0.01
	NNF	0.9231 ± 0.046
	NF	1.2861 ± 0.059
	LF	1.0098 ± 0.009
**DPPH assay**	Ascorbic acid (standard)	0.0505 ± 0.001
	NNF	1.0642 ± 0.051
	NF	1.5641 ± 0.104
	LF	1.0451 ± 0.078

**Table 3:** Contents of total phenolic, flavonoid, and anthocyanin compounds. Data are expressed as means ± SD (*n*= 3).

	Sample (µg/mL)	TPC (GAE µg/mL)	TFC (µg RE/mL)	TAC (µg/mL)
**NNF**	100	3.90 ± 0.60	35.65 ± 6.55	5.57 ± 1.51
	200	7.08 ± 0.70	131.07 ± 2.91	9.69 ± 0.58
**NF**	100	2.80 ± 0.20	1.74 ± 0.26	2.89 ± 1.02
	200	6.22 ± 0.44	49.69 ± 3.84	7.12 ± 0.70
**LF**	100	3.21 ± 0.17	34.68 ± 8.39	3.34 ± 0.58
	200	7.89 ± 1.08	135.43 ± 1.45	7.24 ± 0.19

## Data Availability

Data is contained within the article.
